# From meaning to sound: how word learning shapes non-native speech perception

**DOI:** 10.3389/fpsyg.2025.1620837

**Published:** 2025-09-05

**Authors:** Gabriela Tavares, Yuxin Ge, Susana Correia, Patrick Rebuschat

**Affiliations:** ^1^Centre for Linguistics, School of Arts and Humanities, University of Lisbon, Lisbon, Portugal; ^2^Linguistics Research Centre, NOVA University Lisbon, Lisbon, Portugal; ^3^Department of Linguistics and English Language, Lancaster University, Lancaster, United Kingdom; ^4^LEAD Graduate School & Research Network, University of Tübingen, Tübingen, Germany

**Keywords:** speech perception, vowel discrimination, online perceptual training, cross-situational word learning, statistical learning, Portuguese as a second language

## Abstract

**Introduction:**

Adult learners often struggle to perceive and acquire unfamiliar speech sounds in a second language, especially at the initial stages of learning. Traditional perceptual training methods, such as discrimination tasks, tend to be less effective with beginners, as they rely on low-level acoustic judgments and lack meaningful context. This study investigates whether training with cross-situational word learning (CSWL), a meaning-based learning paradigm, can improve the perceptual discrimination of non-native vowel contrasts.

**Methods:**

Thirty-seven native speakers of Hungarian were trained on eight European Portuguese pseudowords through a single CSWL session involving alternating passive and active learning blocks, feedback, and exposure to multiple native voices. Participants completed identification and discrimination tasks before and after training. Non-native word learning and vowel discrimination were measured before and after training, by means of identification and discrimination tasks, respectively.

**Results:**

Learners achieved above-chance word identification, indicating successful lexical learning. However, improvement in vowel discrimination was contrast-specific: participants improved in three of six contrasts, while performance remained low for the most difficult contrast. Learners also showed lower identification accuracy for pseudowords containing this contrast, and individual discrimination ability was associated with word learning success.

**Discussion:**

These findings highlight that while meaning-based training through CSWL can support early lexical and phonological learning, perceptual challenges remain for difficult contrasts. The study advances our understanding of how word learning and sound perception interact during second language acquisition and demonstrates the potential of lexically grounded approaches for perceptual training at the onset of learning.

## Introduction

It is well established that adult learners often encounter difficulties in perceiving and producing certain non-native (L2) sounds (Archibald, 2021). These challenges are commonly attributed to a range of factors, including first language (L1) influence (Flege and Bohn, 2021), as well as cognitive, socio-affective, and experiential individual differences (Nagle, 2022). Recent studies have increasingly focused on understanding the challenges of L2 phonology, with the aim of refining theoretical models of speech learning to support learners in improving their perceptual and pronunciation skills (Baese-Berk et al., 2025; Darcy et al., 2024; Nagle and Baese-Berk, 2022).

Over the past two decades, L2 perception has garnered growing attention, partly due to the notion that it precedes and models production (Flege, 1995). Although recent reviews suggest a more nuanced relationship between perception and production (Flege and Bohn, 2021; Nagle and Baese-Berk, 2022), and possibly a detrimental effect of production in perception (Baese-Berk et al., 2025), a significant body of research indicates that auditory training enhances perceptual abilities and yields tangible improvements in learners’ production accuracy (Sakai and Moorman, 2018; Uchihara et al., 2024).

Another key reason to investigate L2 perception lies in its critical role in lexical access, a process that involves mapping the speech signal onto stored lexical representations. Research suggests that initial misperceptions of the input—largely due to L1 influence—can shape the content of lexical representations (Cutler et al., 2006; Trofimovich and John, 2011). Specifically, if two similar words are perceived as identical during initial exposure, they may be stored with overlapping phono-lexical representations. While perceptual accuracy does not ensure accuracy of lexical representations, it positively contributes to their development (Darcy et al., 2024). This highlights the importance of refining L2 perceptual abilities, particularly during the early stages of language learning.

A substantial number of studies have documented perceptual training programs implemented with L2 learners across classroom, laboratory, and online settings, all aimed at enhancing learners’ speech perception abilities (see Sakai and Moorman, 2018, for a meta-analysis). Although these studies point to a positive effect of perceptual training in L2 speech, most experiments have focused on learners who have already a considerable knowledge of the L2 (intermediate or advanced proficiency). As a result, little is known about the effects of perceptual training with *ab initio* learners (i.e., learners at the onset of acquisition), particularly for L2 vowel acquisition. Importantly, the onset of learning has been described as an optimal period—a *window of maximal opportunity*—, since it is when learners are most receptive to acquiring new phonological contrasts and build more accurate phonological representations in an L2 (Derwing and Munro, 2014). Consequently, we need to address this gap and develop research focusing on this learning stage.

One major reason for the scarcity of studies with *ab initio* learners is the limited range of suitable training tasks. In 25 out of the 27 studies reviewed by Rato and Oliveira (2023), training involved identification tasks using lexical items—words or pseudowords—which require learners to have some knowledge of L2 vocabulary and/or orthographic conventions. Since *ab initio* learners often lack such knowledge, researchers working with this population frequently rely on discrimination tasks instead. However, evidence supporting the effectiveness of discrimination tasks for improving L2 vowel perception at this early stage remains limited (e.g., Correia et al., 2025; Ge et al., 2025a; Tavares, 2024).

This lack of robust outcomes may reflect the limitations of discrimination tasks themselves. These tasks are often communicatively impoverished, lacking meaningful context, and focus solely on low-level acoustic differences. As a result, they may not effectively support the development of phonological representations. Indeed, recent findings suggest that improvements in phonetic discrimination do not readily transfer to word learning (Ge et al., 2025a). This points to the possibility that phonetic training, while useful, may not be sufficient on its own to help learners map new sounds onto meaningful lexical units—particularly at the onset of learning. Training approaches that incorporate meaning may be more effective in supporting phonological development.

To address the limitations of traditional phonetic training, we propose the cross-situational word learning (CSWL) paradigm as a promising alternative. CSWL offers a meaning-based framework that may foster the acquisition of novel L2 sounds by promoting the formation of robust, contextually grounded phonological representations. Unlike traditional identification tasks, which typically rely on learners’ prior knowledge of L2 vocabulary or orthographic conventions, CSWL requires no such knowledge. Instead, it enables learners to infer word meanings through repeated exposure to ambiguous word–object pairings, gradually mapping sounds to referents based on statistical regularities. This feature makes CSWL particularly well suited for *ab initio* learners, as it embeds unfamiliar sounds in meaningful contexts without requiring explicit instruction or pre-existing lexical representations (Yu and Smith, 2007; Monaghan et al., 2019; Roembke et al., 2023).

### Cross-situational word learning in L2 speech perception

Children can rapidly acquire novel words through exposure to their L1s, without requiring explicit instruction. The learning mechanism underlying this ability—statistical learning—enables them to build vocabulary and grammar by tracking patterns in the distribution of linguistic input across contexts, associating words with referents, and constructing meaning (Williams and Rebuschat, 2022). This capacity has been extensively studied as a foundational cognitive process in language acquisition (Rebuschat, 2022). CSWL refers to a mechanism that mirrors this statistical learning process by modeling how learners disambiguate word meanings through repeated exposure to word–object pairings in referentially ambiguous situations. CSWL has emerged as a valuable experimental paradigm for studying how learners—both children and adults—use statistical regularities to acquire vocabulary in both L1 and L2 contexts (see Roembke et al., 2023, for review).

**FIGURE 1:**
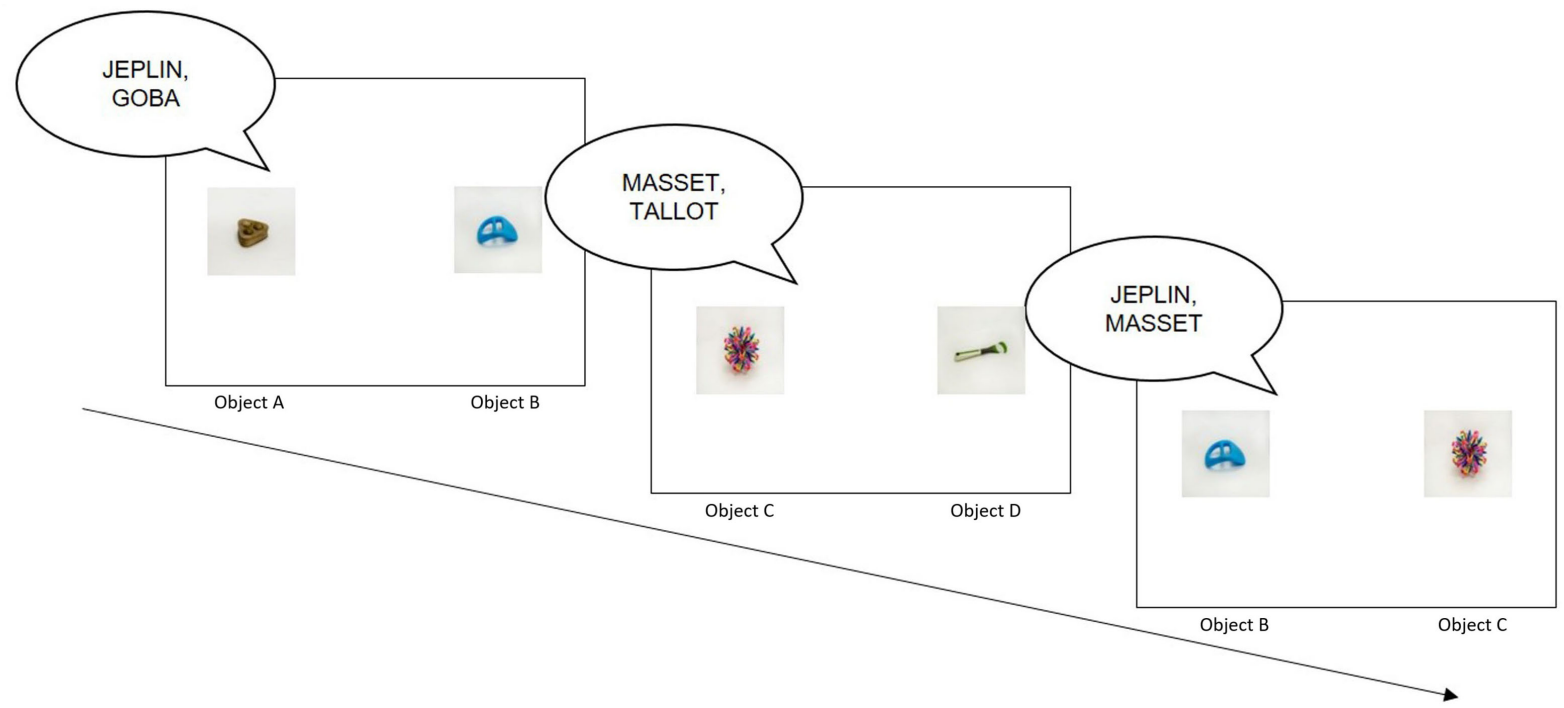
Illustration of a CSWL task following Yu and Smith (2007), adapted from Roembke et al. (2023), © 2023 Roembke, Simonetti, Koch, and Philipp. This is an open-access article distributed under the terms of the Creative Commons Attribution License (CC BY). In the first trial, the participants hear “JEPLIN, GOBA” and see objects A and B, for example. In the second trial, they hear “MASSET, TALLOT” and see objects C and D. In the third trial, they hear “JEPLIN, MASSET” and see objects B and C, and are able to match “JEPLIN” to object B and “MASSET” to object C.

In a typical CSWL experiment, participants are exposed to sequences of learning trials where they hear unfamiliar words while viewing multiple potential referents, without receiving direct feedback. Over successive trials, learners track co-occurrence patterns between words and objects, gradually identifying correct pairings based on statistical consistency. As trials proceed, participants develop increasingly robust associations between specific pseudowords and their intended referents. A well-known example comes from Yu and Smith (2007), in which participants were presented with unfamiliar objects and pseudowords and asked to infer mappings without being told that each word corresponded to a single referent. Despite this ambiguity, participants successfully learned the word–referent pairings, demonstrating the power of statistical learning. Figure 1 illustrates a CSWL task modeled after Yu and Smith (2007).

While early CSWL research primarily employed pseudowords that conformed to the phonotactic constraints of the learners’ L1 (e.g., Yu and Smith, 2007), more recent studies have extended the paradigm to investigate CSWL involving non-native phonological contrasts (e.g., Ge et al., 2025b,2025c). Tuninetti et al. (2020) were the first to explore whether learners could acquire non-native words via CSWL. In their study, native Australian English speakers were exposed to Brazilian Portuguese or Dutch pseudowords, targeting vowel contrasts that were either perceptually easy or difficult based on the learners’ L1. Participants completed a training phase involving trials with two pseudowords and two referents, followed by a test phase requiring word–object matching. Results indicated successful word learning overall, but with diminished performance for pseudowords forming minimal vowel pairs, particularly when perceptual discrimination was more difficult.

A similar study by Escudero et al. (2022) compared Australian English and Mandarin Chinese speakers on their ability to learn English pseudowords as minimal or non-minimal pairs. Participants were exposed to vowel minimal pairs (e.g., /dit/–/dʊt/), consonant minimal pairs (e.g., /bɔn/–/pɔn/), and non-minimal pairs (e.g., /dit/–/bɔn/). While both groups performed above chance across conditions, native English speakers outperformed the Mandarin speakers, particularly in minimal pair learning. Interestingly, Mandarin speakers did not show the expected pattern of greater difficulty with vowel contrasts compared to consonantal contrasts, potentially due to their attentional orientation toward pitch variation in the infant-directed speech used in the stimuli, which may have distracted them from segmental cues.

A more recent study by Ge et al. (2025a) examined whether perceptual training enhances CSWL outcomes. In the first experiment, native English and Portuguese speakers completed a CSWL task using Portuguese pseudowords forming minimal and non-minimal pairs. While both groups succeeded in learning non-minimal pairs, minimal pair learning was significantly more challenging, especially for the non-native group, which performed at chance. In a follow-up experiment, a new group of English speakers was assigned to one of three pre-training conditions: AX discrimination (judging whether two sounds are the same or different), oddity discrimination (identifying the odd token on a sequence), or no training. After this perceptual training, participants again completed the CSWL task. Results showed no significant differences across training groups, and minimal pair learning remained at chance levels, suggesting that the type of perceptual training used did not enhance the ability to learn non-native minimal pairs.

One explanation for the limited minimal pair learning observed in Ge et al. (2025a) may relate to the structure of the CSWL task itself. In their experiment, participants were exposed to a single pseudoword per trial alongside two potential referents, offering limited contrastive input. In contrast, the studies by Tuninetti et al. (2020) and Escudero et al. (2022) presented two pseudowords per trial, encouraging learners to attend to fine-grained phonological differences. This side-by-side contrast may have facilitated the mapping of similar-sounding words to distinct objects. Additionally, Ge et al. (2025a) used a more demanding design, with a larger stimulus set (24 words and referents), compared to the smaller sets used in earlier studies (8–14), potentially increasing memory load and reducing learners’ ability to track word–object pairings.

A central theoretical issue in CSWL research concerns the role of implicit *versus* explicit learning. Two dominant accounts have shaped the current understanding of the mechanism underlying CSWL. According to the propose-but-verify model (Trueswell et al., 2013), learners form a single hypothesis about a word’s meaning and revise it as needed, suggesting a relatively explicit, hypothesis-driven process. In contrast, the gradual statistical model (Roembke and McMurray, 2016) posits that learners maintain multiple hypotheses and adjust them incrementally as evidence accumulates, consistent with implicit learning mechanisms. Recent work suggests these two mechanisms may operate in tandem: while CSWL is primarily driven by statistical learning, the development of explicit hypotheses can support and enhance the learning process, especially under certain task conditions (Monaghan et al., 2019; Rebuschat, 2022).

Experimental evidence supports this interaction between implicit and explicit processes. In Kachergis et al. (2014), participants performed significantly better when they were explicitly informed of the task’s objective—to track co-occurrences or infer word meanings—compared to when no guidance was given. These findings suggest that while implicit learning can occur without instruction, explicit awareness of the task’s goal facilitates performance. Related research on artificial grammar learning and L2 vocabulary acquisition has also demonstrated that explicit instruction enhances learning outcomes, even when the underlying mechanisms are implicit (Goo et al., 2015; Hamrick and Rebuschat, 2014; Monaghan et al., 2019).

Despite the success of CSWL in capturing aspects of early lexical acquisition, the application of this paradigm to L2 learning remains underexplored, particularly for learners at the very beginning of acquisition. Most research on L2 speech learning has focused on intermediate or advanced learners, leaving a gap in our understanding of how *ab initio* learners form phonological representations and acquire novel vocabulary. The limited success of phonetic training methods with naïve learners may be due to their reliance on isolated acoustic cues, which may not be readily integrated into lexical representations without meaningful context (Correia et al., 2025).

Cross-situational word learning offers a promising alternative by coupling sound discrimination with meaning through referential mapping. By embedding non-native sounds in word–referent pairings, CSWL may encourage the formation of phono-lexical representations that support both perception and vocabulary development. This paradigm is particularly valuable for *ab initio* learners, as it does not presuppose prior lexical or semantic knowledge. Instead, it allows learners to build form–meaning associations from the ground up. Unlike traditional explicit word learning paradigms, CSWL does not require direct instruction or conscious memorization of word–referent pairings. Rather, it places learners in situations where they must extract statistical regularities from ambiguous input, closely mirroring the challenges faced by language learners in naturalistic settings.

In terms of cognitive and representational demands, CSWL engages learners’ abilities to track patterns across multiple exposures, requiring attention (Crespo and Kaushanskaya, 2021; Yu et al., 2012) and memory (Li and Benitez, 2025; Neveu and Kaushanskaya, 2023; Vlach and DeBrock, 2017), but without the explicit demands of rote memorization or metalinguistic analysis. While it is less semantically rich than paradigms involving meaningful contexts or narratives, CSWL’s design ensures that learning is lexically grounded. Learners map sounds to referents, yet the focus remains on the phonological form, which is critical for early L2 acquisition. However, findings to date suggest that the potential of CSWL is constrained by factors such as phonological similarity between words (Escudero et al., 2022; Ge et al., 2025c), the complexity of the stimulus set (Ge et al., 2025a), and whether participants receive explicit guidance (Kachergis et al., 2014; Monaghan et al., 2019). Task design features, including the use of minimal or non-minimal pairs, the number of contrasts per trial, and the inclusion of feedback, are likely to modulate learners’ sensitivity to phonological distinctions.

A further theoretical strength of CSWL lies in its position at the intersection of implicit and explicit learning mechanisms. CSWL is not purely implicit or explicit but rather allows for the interaction of both processes. While statistical learning mechanisms are primarily implicit, recent research suggests that explicit hypothesis formation can support and enhance learning, especially under certain task conditions. This dual-process nature makes CSWL a powerful tool for investigating the mechanisms underlying L2 phonological learning, offering unique insights that are not easily captured by more explicit or semantically rich word learning paradigms.

In summary, CSWL has emerged as a powerful tool to investigate the mechanisms underpinning word learning in both native and non-native language contexts. The paradigm’s relevance extends to fundamental questions in the psychology of language, including how learners integrate auditory and visual information, how perceptual difficulty interacts with lexical development, and how statistical learning interacts with cognitive control and awareness (Rebuschat, 2013, 2022). In the present study, we investigate the potential of CSWL as a training tool for L2 phonological acquisition in learners at the earliest stages of exposure to the target language. While previous research has primarily employed CSWL to observe acquisition processes, our approach explores its effectiveness as an active training method. To this end, and in line with its use as a training instrument, we address two important gaps in CSWL research: the inclusion of feedback and the incorporation of speaker variability. Feedback is well established in L2 learning as a critical factor for enhancing linguistic accuracy and learner engagement (Li, 2010; Monaghan et al., 2021). Notably, Monaghan et al. (2021) represents a rare exception in CSWL research for introducing explicit feedback; in their study with native English speakers exposed to an artificial language, the authors found that explicit feedback facilitated learning. Regarding speaker variability, although this is a common feature in phonetic training studies (Thomson, 2018), it is rarely present in CSWL research (see Crespo et al., 2023; Crespo and Kaushanskaya, 2025, for notable exceptions).

### The present study

This study is the first in a larger project that systematically investigates the relationship between word learning and perceptual discrimination. In this initial phase, we examine whether learning novel words that contain non-native vowels can enhance learners’ ability to perceptually discriminate those same vowels. Our broader aim is to explore a methodological approach in which perceptual training is meaning-based and lexically grounded, providing a more ecologically valid alternative to traditional discrimination tasks.

To this end, we developed a novel training procedure that integrates two types of CSWL tasks: a *passive* learning version, modeled after Yu and Smith (2007), and an *active* learning version, based on Monaghan et al. (2019). Hungarian native speakers were trained on European Portuguese pseudowords across both tasks. Our CSWL design incorporated two key innovations: feedback, to ensure high levels of word learning accuracy, and speaker variability, in line with phonetic training studies. Including multiple voices aimed to promote more robust phonological category formation, especially given the variability learners are likely to encounter in real-world input.

This study addressed four interrelated research questions, each grounded in theoretical and empirical insights from L2 speech learning.

The first question asked whether learning non-native words through CSWL could improve learners’ ability to discriminate non-native vowel contrasts. We predicted that mapping novel words to referents in a meaningful context would promote the development of phonological representations, leading to measurable improvements in vowel discrimination. This effect was expected to be particularly strong for contrasts involving vowels absent from the learners’ L1, as these require the formation of entirely new categories (Flege and Bohn, 2021).

The second question examined whether learners would have greater difficulty acquiring pseudowords that contained harder-to-discriminate vowels. We anticipated that perceptual similarity would constrain lexical learning, such that pseudowords including the confusable vowels /ε/ and /e/—which are known to be particularly challenging for Hungarian listeners (Tavares et al., 2024)—would be learned less successfully. In contrast, pseudowords containing vowels that are more distinct to these listeners, such as /ε/ and /-ɨ/, were expected to be identified more accurately.

The third question focused on the viability and effectiveness of CSWL as a training method for learners at the onset of L2 acquisition. We expected that even *ab initio* learners with no prior exposure to the target language would be able to acquire novel pseudowords through a single CSWL session. Specifically, we predicted that participants would perform significantly above chance both during training and in the post-training identification task, demonstrating that CSWL is a feasible and effective method for supporting early L2 lexical and perceptual learning.

Finally, we asked whether learning outcomes in both word learning and discrimination tasks would vary depending on the perceptual difficulty of the vowel contrasts. We anticipated that pseudowords containing perceptually easier contrasts—such as /ε/–/-ɨ/—would be learned and discriminated more successfully than those containing more challenging contrasts, particularly /ε–/e/. These contrast-specific effects were expected to highlight the influence of perceptual salience on the outcomes of CSWL-based learning.

## Materials and methods

Our study follows a pre-test/post-test design, in which we trained Hungarian native speakers with European Portuguese (henceforth: Portuguese) pseudowords containing four vowels: /ɐ/,/ɛ/,/e/, and/ɨ/. These vowels were selected based on perceptual difficulties observed in a previous study (Tavares et al., 2024). While /ɐ/and/ɨ/ are absent from the Hungarian vowel inventory, /ε/ and /e/ are part of the Hungarian vowel space (Markó, 2017). However, the experiments conducted in the above-mentioned study showed that the Portuguese /ε/ is often perceived as a Hungarian deviant /eː/, and, even when identified as /ε/, it is perceived as a deviant form of the Hungarian category. Moreover, the Portuguese /ɐ/–/ɛ/ contrast often forms a single-category assimilation (Best, 1995), as both vowels are systematically categorized into /ε/ in Hungarian. Consequently, Hungarian listeners show difficulties in discrimination of /ε/–/e/ and /ɐ/–/ɛ/. As for /-ɨ/, results showed categorization difficulties for this vowel, with accuracy below 40%.

As mentioned, training included two types of CSWL tasks, a *passive* task and an *active* task. Feedback was introduced only in the latter. In the *passive* task, participants were presented auditorily with two pseudowords and visually with the corresponding two nonsense objects and were asked to learn the pseudowords and the correspondent nonsense object, just by listening and observing. In the active CSWL task, participants practiced what they learned before: they were presented with an auditory token of a pseudoword and visually with two nonsense objects and had to decide which object corresponded to the word they heard.

Before and after the training, we assessed learners’ ability to discriminate the target Portuguese vowels using an AXB task. Both the pre- and post-test sessions also included a lexical identification task to evaluate whether participants had learned the non-native pseudowords through CSWL training. Additionally, prior to the pre-test, participants completed a brief sociolinguistic questionnaire.

All tasks—consent form, questionnaire, training and tests—were created and hosted online in Gorilla Experiment Builder (Anwyl-Irvine et al., 2020). Instructions were presented in the L1 of the participants (Hungarian).

The study was reviewed and approved by the Faculty of Arts and Social Sciences and Lancaster Management School’s Research Ethics Committee. Materials, anonymized data and R scripts are available at https://osf.io/ey8tf/?view_only=412353eda8f24bc6ba0be3bdad58b60f. Preregistration can be accessed at https://osf.io/vn7tb/?view_only=f3f4d671052a40948dcd0b9fd78cf536.

### Participants

Participants were recruited via Prolific, receiving 9 GBP per hour upon completion of all the tasks. Thirty-eight Hungarian native speakers completed the experiment. All participants reported no previous experience of learning or prolonged contact with Portuguese, and no language disorders or trauma and normal or corrected-to-normal vision and hearing. One participant reported being Hungarian-English bilingual and was thus excluded from data analysis. The included participants were aged 21–47 (mean = 30.4, SD = 7.1), and 17 were female speakers. All participants reported knowledge of L2 English. Twenty-six participants indicated advanced proficiency and daily use of English, while ten reported intermediate proficiency, also with daily or frequent use. One participant reported beginner-level proficiency, using English only a few times a year. Other reported languages included Danish, Dutch, French, German, Italian, Japanese, Korean, Pali, Spanish, Swedish, and Vietnamese, all spoken at beginner or intermediate levels.

### Materials

#### Words (auditory stimuli)

We selected eight Portuguese pseudowords from Tavares (2024), targeting four Portuguese vowels: /ɐ/,/ɛ/,/e/, and /-ɨ/. The Portuguese vowels were inserted in two consonant contexts, /gV/ and/zV/. The choice of using two CV contexts was threefold. First, the use of a monosyllabic structure is crucial to avoid word stress, since Hungarian speakers experience perceptual difficulties with this feature (Honbolygó et al., 2017). Furthermore, research has shown that open syllables are more effective than pseudowords in improving L2 vowel perception (Thomson and Derwing, 2016). Secondly, none of the resulting tokens convey meaning in Hungarian, thereby minimizing semantic interference. Thirdly, the selected consonants, /g/ and /z/, differ significantly in both place and manner of articulation. We expect that, similar to how speaker variability contributes to categorical processing, variability in phonetic context will also contribute to categorical processing rather than phonetic discrimination.

The tokens—/ɡɐ/,/ɡɛ/,/ɡe/,/ɡɨ/,/zɐ/,/zɛ/,/ze/, and /z-ɨ/—were produced by two female speakers of Portuguese from the dialectal area of Lisbon. Stimuli recording and preparation, as carried out by Tavares (2024), were as follows: Tokens were produced within carrier sentences to ensure consistent vowel quality and intonation. For the recordings, a TASCAM DR-05 V2 digital recorder and a Beyerdynamic MCE 85 BA condenser microphone were employed. The audio was saved in .wav format, with a sampling frequency of 44,100 Hz, mono channel, and 32-bit depth. Each recording was individually edited using Audacity (Audacity Team, 2020) to remove background noise. Special care was taken to normalize vowel duration, given the demonstrated sensitivity of Hungarian listeners to vowel length (Markó, 2017). Vowel duration manipulation was performed using Praat (Boersma and Weenink, 2020). Finally, the mean intensity throughout each syllable was equalized across all tokens to 70 dB.

#### Novel objects

**FIGURE 2:**
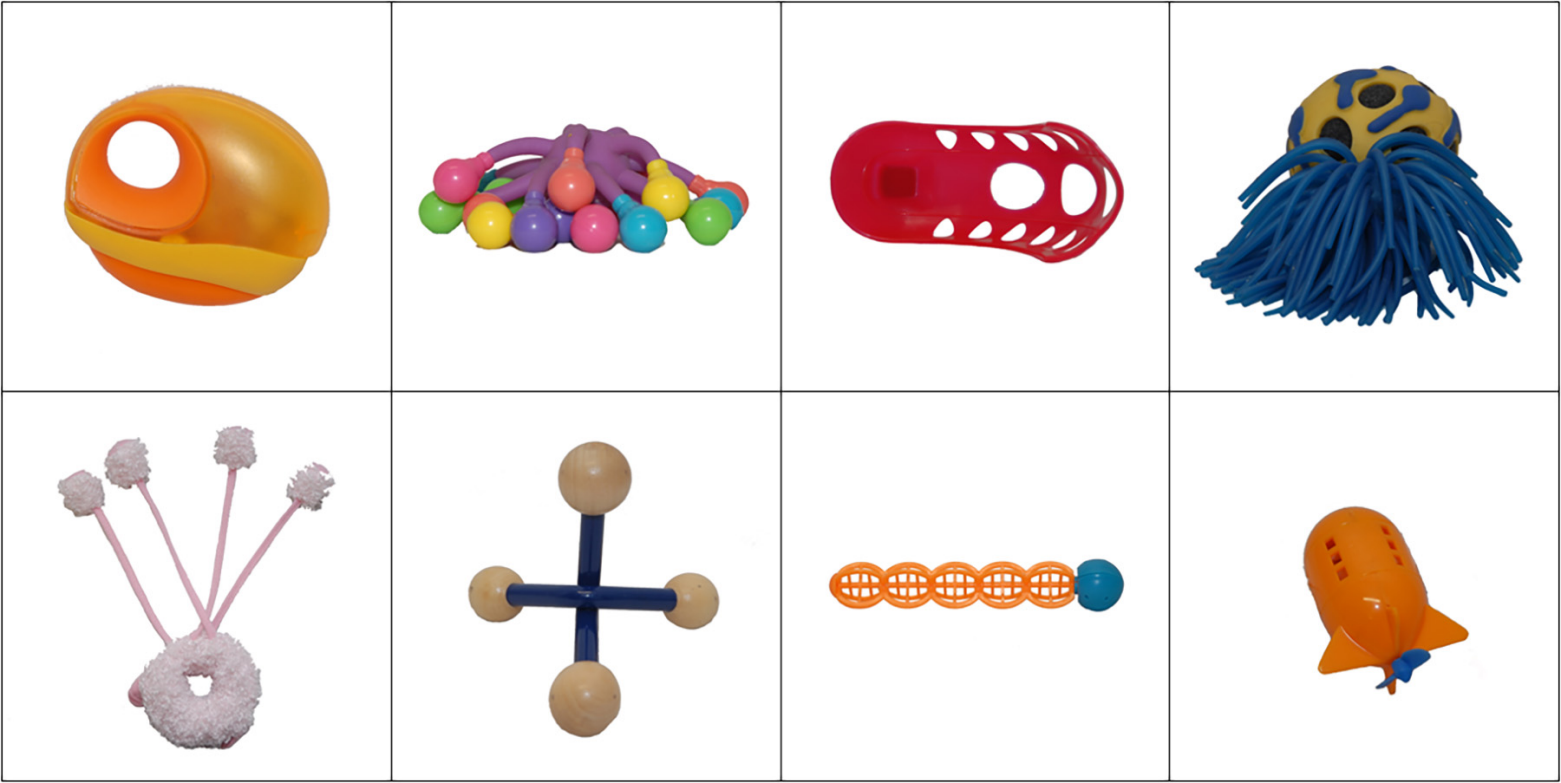
Novel objects selected from the NOUN Database (Horst and Hout, 2016).

Each of the eight pseudowords was randomly mapped onto one of eight novel objects from the NOUN Database (Horst and Hout, 2016; Figure 2). To control for memory and learning effects of a particular mapping over others, we created four different word-object mappings, displayed in Supplementary material.

### Tasks

To address our research questions, we employed three tasks: an AXB discrimination task, a lexical identification task, and a CSWL task. The CSWL task served as our training instrument, while the AXB discrimination and lexical identification tasks functioned as pre- and post-training assessments. Comparing AXB pre- and post-test results enables us to evaluate changes in discrimination abilities following CSWL training (Research Question 1, RQ1). The lexical identification test assesses whether word learning occurred as a result of the CSWL training (RQ3). By analysing performance across both testing tasks, we can further examine the impact of perceptual difficulties on word learning (RQ2) and explore the relationship between perception and word learning (RQ4).

#### Cross-situational word learning tasks

Throughout the CSWL, both in the *passive* and the *active* CSWL trials, participants were always presented with two objects on the screen, corresponding to two pseudowords. These doublets were created in such a way that each word/object would occur with all other words/objects, in a total of 28 possible combinations. The combinations included twelve doublets that configured vowel minimal pairs, (e.g., /ɡɐ/−/ɡɛ/,/zɐ/−/zɛ/), four doublets that configured consonant minimal pairs (e.g., /ɡɐ/−/zɐ/,/ɡɛ/−/zɛ/), and 12 doublets that configured non-minimal pairs (e.g., /ɡɛ/−/zɐ/,/ɡɐ/−/zɛ/). Although the aim was the perception of vowel contrasts, the inclusion of consonant minimal pairs and non-minimal pairs increased the co-occurrences of a word/object with other words/objects. The 28 contrasts are listed in Supplementary material.

In the *passive* CSWL trials, participants were presented with two pseudowords auditorily and corresponding nonsense objects visually, learning the associations through listening and observation. The presentation of pseudowords and objects followed either a congruent or incongruent order, as exemplified in Table 1. In the *congruent* order, participants heard the sequence *word A−word B* and the object A appears on the left and the object B, on the right. In the *incongruent* order, participants hear the sequence word A−word B, the object A appears on the right and the object B, on the left.

**TABLE 1:** Combinations for the /ɡɐ/−/ɡɛ/ contrast, for the *passive* CSWL task.

First stimulus	Second stimulus	Object on the left	Object on the right	Condition
/ɡɐ/ speaker 1	/gε/ speaker 2	A	B	Congruent
/ɡɐ/ speaker 1	/gε/ speaker 2	B	A	Incongruent
/ɡɐ/ speaker 2	/gε/ speaker 1	A	B	Congruent
/ɡɐ/ speaker 2	/gε/ speaker 1	B	A	Incongruent
/gε/ speaker 1	/ɡɐ/ speaker 2	A	B	Incongruent
/gε/ speaker 1	/ɡɐ/ speaker 2	B	A	Congruent
/gε/ speaker 2	/ɡɐ/ speaker 1	A	B	Incongruent
/gε/ speaker 2	/ɡɐ/ speaker 1	B	A	Congruent

The *passive* CSWL task included 224 trials: 28 doublets (12 vowel contrasts + 4 consonant contrasts + 12 non-minimal pairs) × 8 combinations (2 orders of speaker (first or second) × 2 positions of objects (right or left) × 2 conditions word/object (congruent or incongruent)).

In the *active* CSWL trials, although participants were presented visually with two objects in each trial, they only heard one word, corresponding to one of the two objects. In these trials, participants were asked to choose which object matched the word they heard and received feedback on their responses. We used an emoji to avoid orthographic interference (“smiley face” = correct answer, “sad face” = incorrect). In case of an incorrect answer, participants were immediately presented with the same trial. No limit of attempts was set. The *active* CSWL task included 224 trials: 8 target pseudowords × 2 speakers × 2 target position × 7 foils. Table 2 exemplifies the trials for the pseudoword /ɡɐ/.

**TABLE 2:** Combinations for the word /ɡɐ/, for the *active* CSWL task.

Stimuli	Object on the left	Object on the right
/ɡɐ/ speaker 1	A	B
/ɡɐ/ speaker 1	B	A
/ɡɐ/ speaker 2	A	B
/ɡɐ/ speaker 2	B	A

The 224 *passive* trials and the 224 *active* trials were equally divided into 4 blocks, each with 56 trials. The occurrence of each trial type was balanced between blocks. For example, each block had the same number of vowel minimal-pair contrasts, consonant minimal pair contrasts and non-minimal pair contrasts. The order of the Portuguese speakers was also counterbalanced, as well as the occurrence of each object on the left and on the right. In Supplementary material we describe the conditions across blocks in each CSWL task.

**FIGURE 3:**
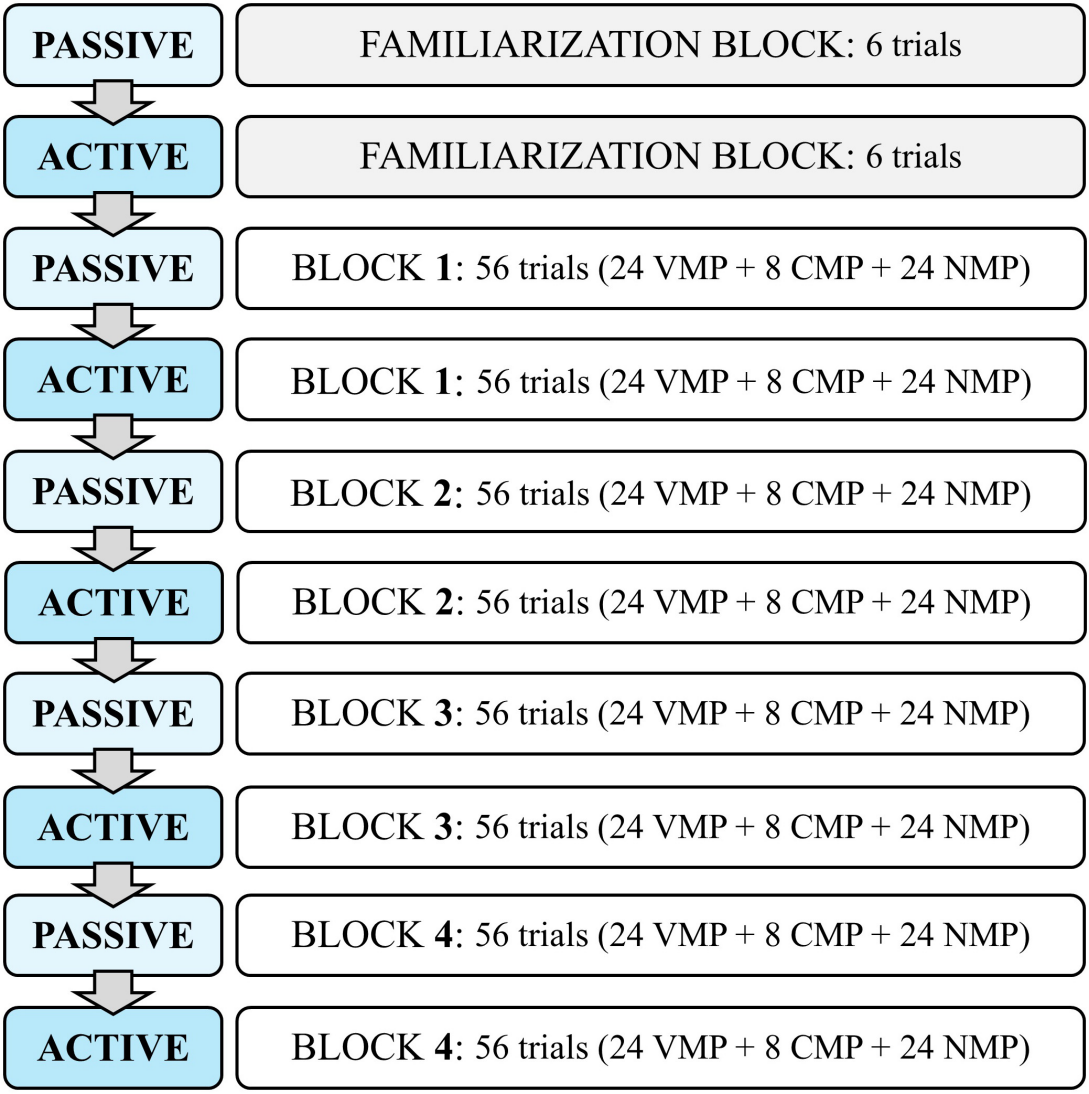
Structure of the CSWL task: passive and active blocks’ sequence, with the number of trials by contrast type—vowel minimal pairs (VMP), consonant minimal pairs (CMP) and non-minimal pairs.

The *passive* and *active* CSWL blocks were alternately presented in a single training session (*passive*→ *active*→ *passive*→ *active*→ *passive*→ *active*→ *passive*→ *active*; Figure 3).

Before the main trials, participants completed a familiarization phase, with trials similar to the main trials, but with L1 real words and real objects. Since monosyllabic nouns are rare in Hungarian, and this could hinder the perception of monosyllabic words as nouns, in the familiarization task, we selected monosyllabic Hungarian nouns: /fεj/ ‘head’, /fyː/ ‘grass’, /tεj/ ‘milk’, and /tyː/ ‘needle’. Tokens were recorded by two female Hungarian native speakers.

The inter-stimulus interval (ISI) was set to 1200 ms. All trials were randomized between participants, within each block. Furthermore, a 10-s break was inserted in the middle of each block.

**FIGURE 4:**
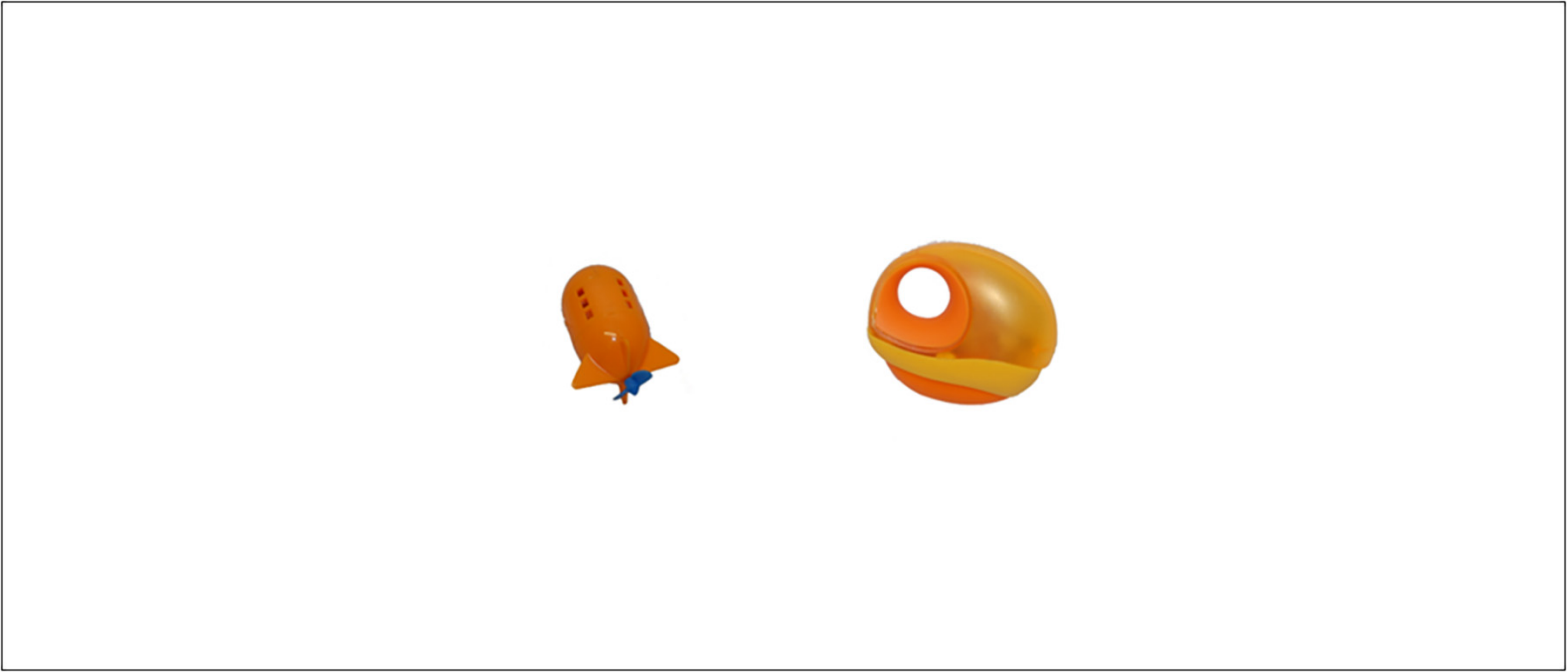
Computer screen exemplifying the CSWL tasks. In the *passive* trials, participants heard a sequence of two pseudowords, each one corresponding to one of the novel objects on the screen. In the *active* trials, participants hear one pseudoword, corresponding to one of the novel objects on the screen, and had to decide which was the object.

Figure 4 displays a computer screen for a CSWL trial.

#### Lexical identification test

**FIGURE 5:**
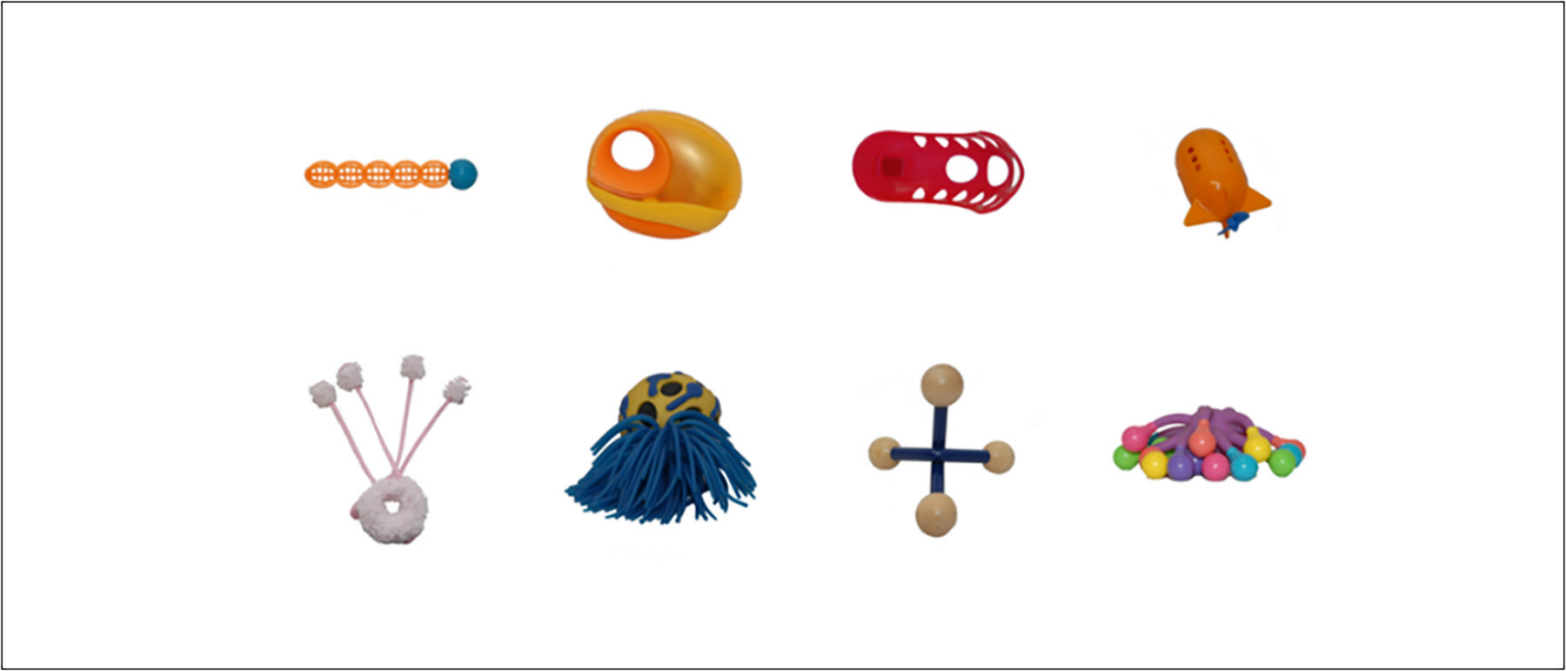
Computer screen of the lexical identification task. In this task, participants heard one pseudoword, corresponding to one of the novel objects on the screen, and had to decide which was the object.

This test was designed to assess if participants learned the Portuguese pseudowords; it was administered twice, once as pre-test and once as post-test, each with different trial randomizations. Although we anticipated that participants would primarily be guessing the answers at pre-test, we assessed word knowledge prior to any learning to determine whether participants held any pre-existing assumptions about specific word-picture mappings based on characteristics such as sound, shape or color. The test consisted of a forced-choice task, in which participants heard the pseudowords and had to decide, on a trial-by-trial basis, which object (displayed in a 4 × 2 grid) was associated with the presented pseudoword. No feedback was provided. The task included 48 trials, corresponding to the 8 pseudowords × 2 speakers × 3 repetitions. A 10-s pause was inserted in the middle of the task. The position of the objects was randomized in each trial, and trials were randomized between participants. Figure 5 displays a computer screen for one trial of the identification task.

#### AXB discrimination test

In this task, we tested participants’ ability to discriminate the Portuguese vowels. This was also used twice, once as a pre-test and once as a post-test, with trial sequences randomized. In each trial, participants were auditorily presented with three pseudowords and had to decide if the first (A) or the third word (B) was similar to the second one (X). For every trial, A and B were produced by one speaker, and X was produced by a different speaker. In other words, participants always had to match tokens from two different speakers. The order of the speakers was counterbalanced across trials.

Since the aim was to test vowel discrimination, we only presented vowel minimal pairs (e.g., /ɡɐ/−/ɡɛ/,/zɐ/−/zɛ/). For each contrast, 8 trials were created: 2 contexts (/gV/,/zV/) × 2 speakers × 2 identities of X (A or B). The AXB task included 192 trials, corresponding to 12 contrasts × 8 trials × 2 repetitions. In line with the CSWL task, we set the ISI to 1200 ms. To avoid fatigue effects, the 192 trials were divided into 6 blocks, separated by 10-s breaks.

Similar to the CSWL tasks, a brief familiarization phase was included, using the Hungarian tokens /fεj/, /tεj/, /fyː/, and /tyː/. Trials were randomized between participants.

**FIGURE 6:**
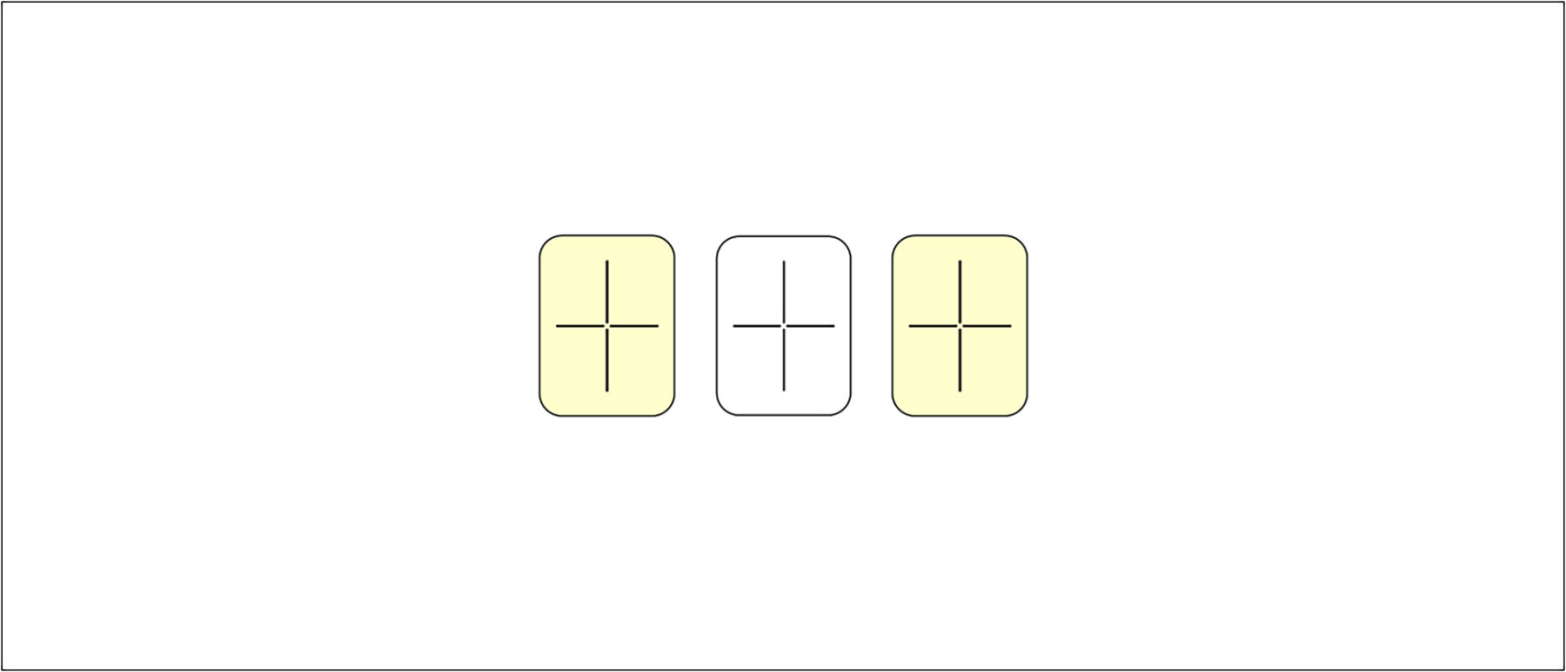
Computer screen of an AXB trial. In this task, participants heard a sequence of three pseudowords and had to decide whether the first (A; rectangle on the left) or the third word (B; rectangle on the right) was more similar to the second word (X; rectangle in the middle).

Figure 6 displays the computer screen for an AXB trial.

### Procedure

**FIGURE 7:**
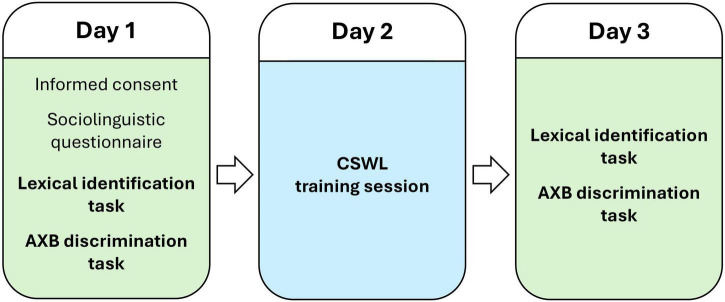
Timeline of the experiment, with the conducted tasks.

The experiment consisted of three sessions, administered on consecutive days (Figure 7), and for each session, participants were directed from Prolific to the experimental platform (Gorilla), to complete the tasks. In the first session (Day 1), participants provided informed consent, completed the sociolinguistic questionnaire and the pre-tests, that is, the AXB discrimination task and lexical identification task (ca. 30 min total). In the second session (Day 2), participants completed the CSWL training (ca. 40 min total). They were randomly assigned to one of the four mapping groups, that is, to one of the counterbalanced versions of word-object pairings. This randomization was balanced, so that the groups would have a similar number of participants. In the third session (Day 3), participants completed the post-test, which again consisted of the lexical identification task followed by the AXB discrimination task (ca. 25 min total). By administering the post-test on a separate day from the training session, our goal was to assess retention of learning, which provides a more robust measure of lasting effects.

Although no time limit was imposed, participants were instructed to respond as quickly and accurately as possible.

### Data analysis

Data analysis followed the procedures outlined in the preregistration and proceeded as follows. To ensure data quality, we applied the following exclusion criteria. First, we searched the data to identify individual responses longer than 30 s, since it was likely participants were not fully engaged with the task during those trials. Our analysis revealed no responses exceeding this threshold. Further, in the CSWL and AXB tasks, we looked for data exhibiting response patterns suggestive of inattentiveness. Specifically, we looked for cases when participants consistently (≥90%) selected the same response option (left or right object; A or B) or showed a repetitive alternating pattern (left/right/left/right/…; A/B/A/B/…). One participant exhibited a repetitive alternating pattern in the first block of the CSWL active trials, leading to the exclusion of these data from the analysis.

Regarding the CSWL task, we analyzed data from the *active* blocks of training. In these tasks, participants received feedback, and, in case of incorrect answer, they had to repeat the trial. Although there was no limit on the number of attempts, participants rarely repeated a trial more than once. Therefore, we considered only the first response to each trial: correct = 1 and incorrect = 0. Responses in the identification and the AXB tests were also coded as correct = 1 or incorrect = 0.

We used generalized linear mixed effects models (GLMER functions, lme4 package; Bates et al., 2015). The statistical significance of fixed effects was determined through a series of ANOVA tests on log-likelihood, comparing models with the fixed effects to a baseline null model containing only random effects. Furthermore, to obtain additional insights into the models’ results, we conducted pairwise comparisons of least-squared means using the EMMEANS function (emmeans package; Lenth et al., 2025), with Bonferroni corrections applied to adjust *p*-values for multiple comparisons. In addition, one-sample Wilcoxon signed-rank tests were used to determine whether word identification accuracy was significantly above chance level in the CSWL task and in the identification post-test.

Anonymized data and R scripts are available at https://osf.io/ey8tf/?view_only=412353eda8f24bc6ba0be3bdad58b60f. Summarized results for each task can be found in Supplementary material.

## Results

### Performance on the training task

**FIGURE 8:**
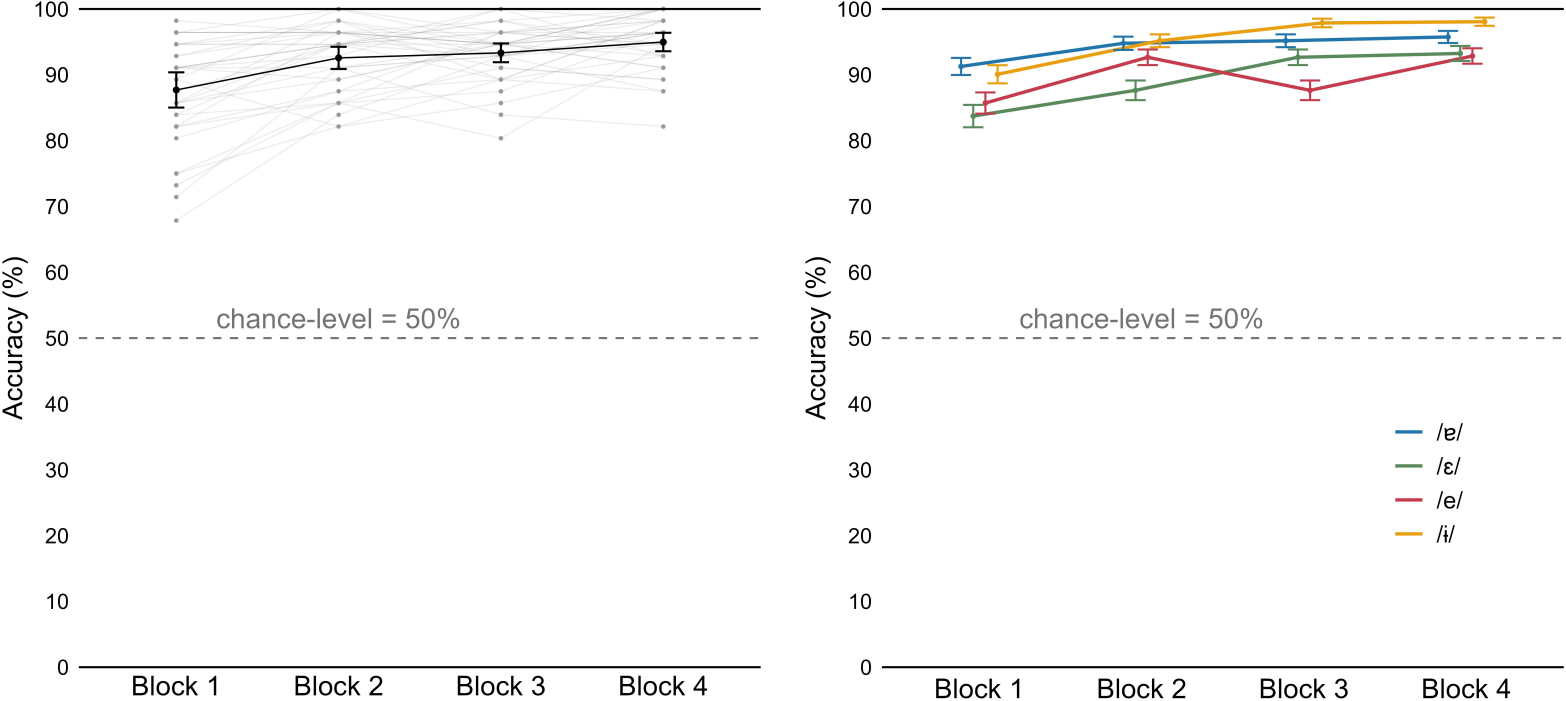
Accuracy for the CSWL active trials, by block. (Left) Mean values by participant (gray) and group mean (black). (Right) Mean group values as a function of vowel. Error bars represent 95% Confidence Intervals.

Figure 8, on the left, displays individual and group mean accuracy values obtained for each block of the *active* CSWL task. The passive version does not require an overt response from participants, and therefore the learning trajectory cannot be plotted. In the active task, participants performed above chance (50%) from the first training block. Mean accuracy across participants further improved from 87.2% (in the first block) to 95.0% (in the last block). One-sample Wilcoxon signed-rank tests conducted for each word for the results of the 1st block, revealed that mean accuracy was significantly above chance in all cases (*p* < 0.001 in all pseudowords). The results clearly demonstrate that participants achieved high accuracy in pseudoword identification.

We constructed a series of linear mixed effect models to investigate progress as a function of vowel (Figure 8, on the right). The ANOVAs on these models revealed that adding *block* as fixed effect to the baseline model improved model fit (χ^2^(1) = 7.532, *p* = 0.006), as well as adding vowel (χ^2^(2) = 16.088, *p* < 0.001). The *block* × *vowel* interaction improved model fit further (χ^2^(4) = 33.123, *p* < 0.001; fixed effects summarized in Table 3), indicating a different improvement rate between the vowels. Pairwise comparisons revealed significant improvement between consecutive blocks for the vowels /-ɨ/ and /ε/ (*p* < 0.001 in all comparisons for both vowels).

**TABLE 3:** Fixed effects for the best fitting model for accuracy in the CSWL tasks, testing vowel effect.

Fixed effects	Estimate	SE	*Z*	*p*
(Intercept)	2.878	0.364	7.905	<0.001***
Block	0.116	0.118	0.988	0.323
Vowel/e/	−1.033	0.348	−2.970	0.003**
Vowel/ε/	−1.550	0.334	−4.641	<0.001***
Vowel/-ɨ/	−1.102	0.417	−2.640	0.008**
Block:vowel/e/	0.056	0.125	0.451	0.652
Block:vowel/ε/	0.274	0.121	2.254	0.024*
Block:vowel/-ɨ/	0.696	0.168	4.147	<0.001***

Number of observations: 8232, Participants: 37. AIC = 4288.9, BIC = 4597.6, log-likelihood = −2100.5. R syntax: glmer(accuracy ∼ block × vowel + (1 + block + vowel + block × vowel | participant_ID), family = binomial, data = CSWL, glmerControl(optCtrl = list(maxfun = 2e5), optimizer = “nloptwrap”, calc.derivs = FALSE)). Reference level: /ɐ/, block 1.

*p ≥ 0.05;

**p ≥ 0.01;

***p ≥ 0.001.

### Performance on the pre-tests and post-tests

#### Lexical identification tests

**FIGURE 9:**
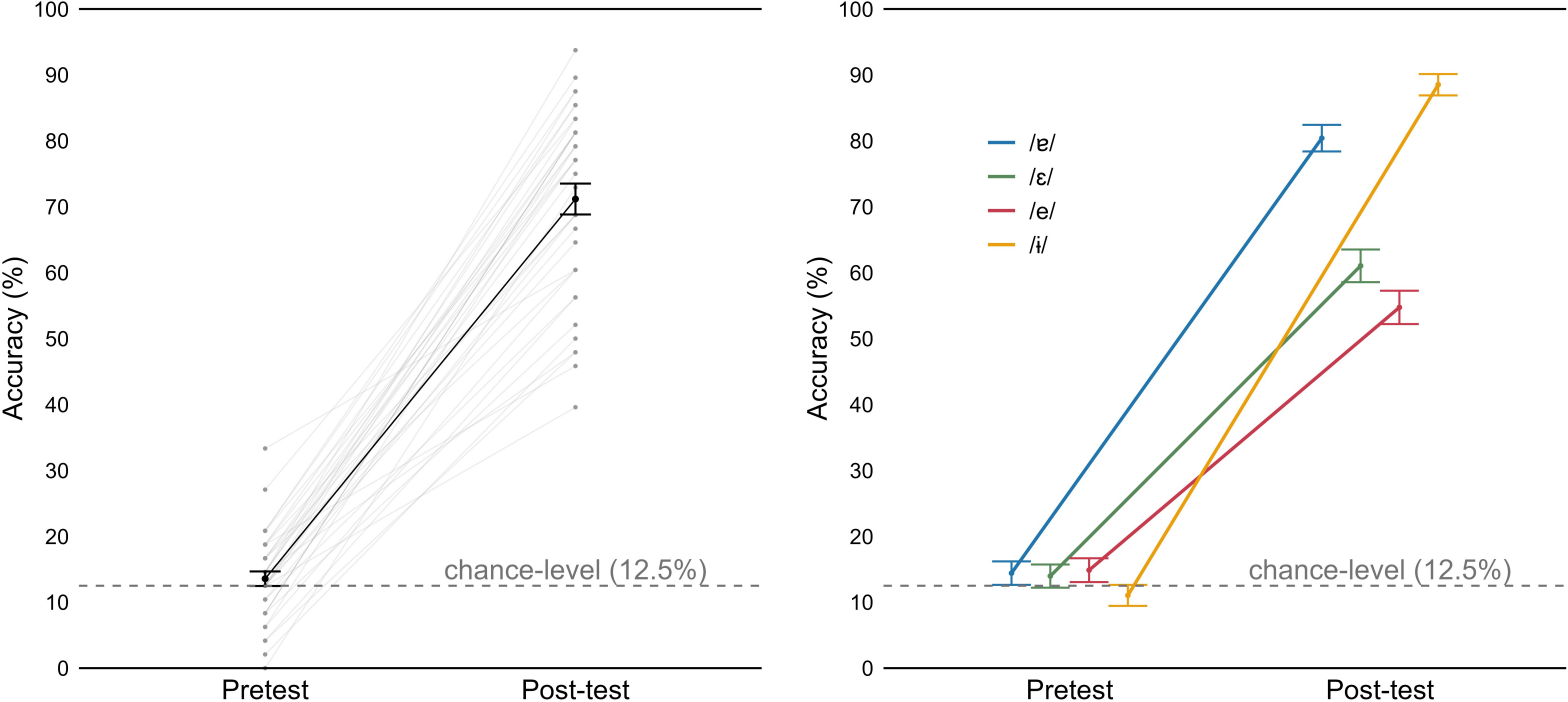
Accuracy for the lexical identification task, in pre-test and post-test. (Left) Mean values by participant (gray) and group mean (black). (Right) Mean group values as a function of vowel. Error bars represent 95% Confidence Intervals.

Individual and group mean accuracy, in pre-test and post-test, is presented in Figure 9, on the left side. In general, all participants performed above chance level (12.5%) in the post-test. Mean accuracy across participants improved from 13.57% in the pre-test to 71.17% in the post-test. One-sample Wilcoxon signed-rank tests showed that while performance on the pre-test was at chance for each word (*p* = 1 in all cases), post-test accuracy was significantly above chance at the post-test (*p* < 0.001 in all cases). These results confirm that word learning took place.

Analysis with the mixed effect models revealed that adding the fixed effect of test significantly improved model fit (χ^2^(1) = 37.122, *p* < 0.001), but not adding vowel (χ^2^(2) = 0.000, *p* = 1). However, the *test* × *vowel* interaction improved the model fit (χ^2^(4) = 94.431, *p* < 0.001; fixed effects summarized in Table 4). The pairwise comparisons revealed significant improvements from pre-test to post-test across all vowels (*p* < 0.001 in all cases). Importantly, in the post-test, accuracy for pseudowords with /-ɨ/ or /ɐ/ was higher than for pseudowords with /ε/ or /e/ (*p* < 0.001 in all comparisons). Furthermore, difference in mean accuracy between pseudowords with /-ɨ/ and pseudowords with /ɐ/ also reached significance (*p* = 0.019).

**TABLE 4:** Fixed effects for the best fitting model for accuracy in the lexical identification tasks, testing vowel effect.

Fixed effects	Estimate	SE	*Z*	*p*
(Intercept)	−2.175	0.245	−8.875	<0.001***
Vowel/e/	0.167	0.365	0.457	0.647
Vowel/ε/	−0.086	0.356	−0.241	0.810
Vowel/-ɨ/	−0.367	0.362	−1.013	0.311
Testpost-test	3.916	0.295	13.288	<0.001***
Vowel/e/:testpost-test	−1.694	0.375	−4.521	<0.001***
Vowel/ε/:testpost-test	−1.099	0.345	−3.187	0.001**
Vowel/-ɨ/:testpost-test	1.451	0.382	3.796	<0.001***

Number of observations: 3552, Participants: 37. AIC = 3210.9, BIC = 3482.6, log-likelihood = −1561.4. R syntax: glmer(accuracy ∼ vowel × test + (1 + test + word + test × word | participant_ID), family = binomial, data = ID, glmerControl(optCtrl = list(maxfun = 2e5), optimizer = “nloptwrap”, calc.derivs = FALSE)). Reference level: /ɐ/, pre-test.

**p ≥ 0.01;

***p ≥ 0.001.

#### AXB discrimination tests

**FIGURE 10:**
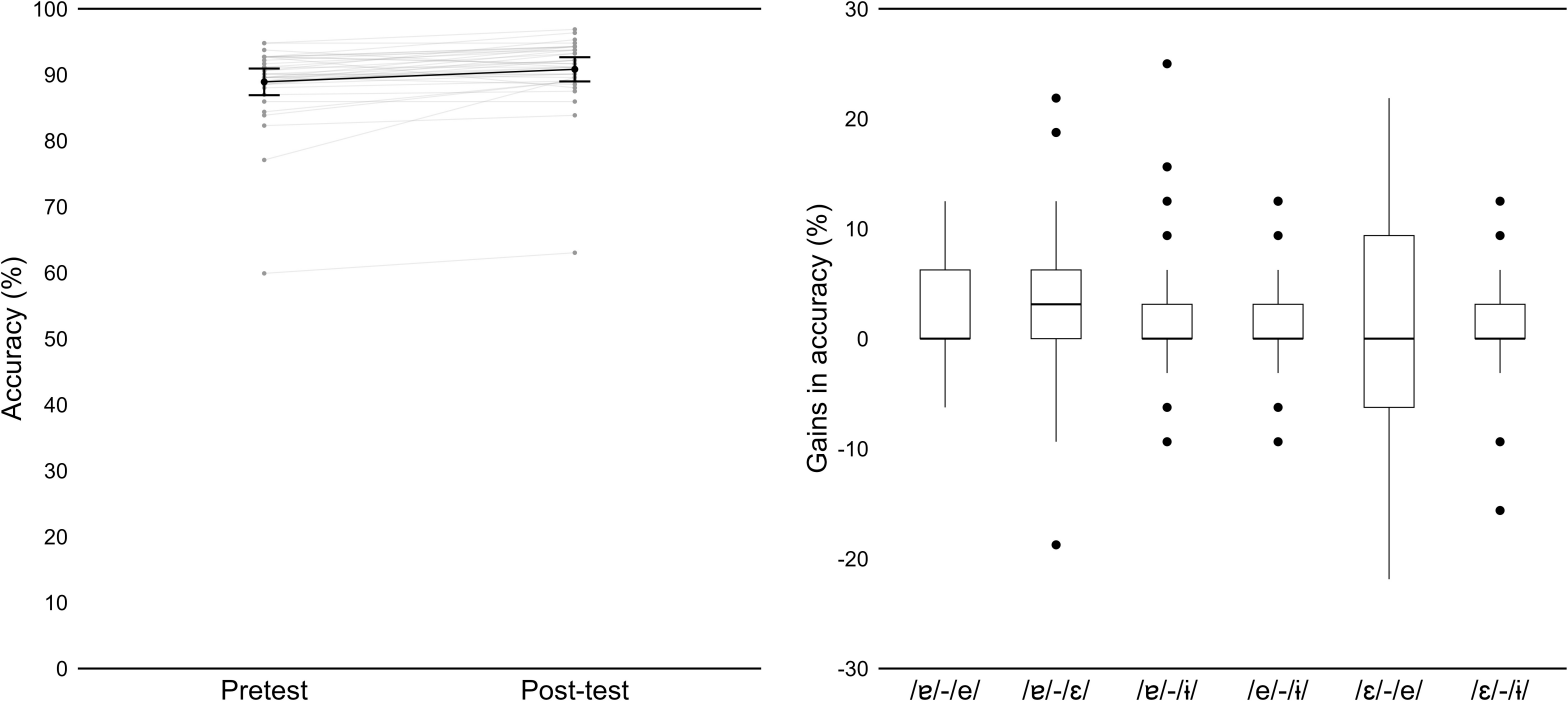
(Left) Mean accuracy for the AXB task, in pre-test and post-test, by participant (gray) and group (black). Error bars represent 95% Confidence Intervals. (Right) Gains in accuracy from pre-test to post-test, as a function of contrast.

Except for one participant, Hungarian listeners showed a high level of accuracy already at pre-test (Figure 10, on the left). Mean accuracy across participants improved from 88.9% in pre-test to 90.8% in post-test. Analysis of the mixed effects models revealed no effect of test (χ^2^(1) = 0.071, *p* = 0.7903), but adding vowel contrast to the baseline model improved model fit (χ^2^(4) = 69.064, *p* < 0.001), as well as *test* × *vowel contrast* interaction (χ^2^(6) = 30.000, *p* < 0.001; fixed effects summarized in Table 5). Pairwise comparisons revealed a significant improvement in accuracy for three contrasts: /ɐ/−/e/ (*p* = 0.004), /ɐ/−/ε/ (*p* < 0.001) and /ɐ/−/ε/ (*p* < 0.001). Figure 10, on the right, displays gains in accuracy, from pre- to post-test, for each contrast.

**TABLE 5:** Fixed effects for the best fitting model for accuracy in the AXB tasks, testing vowel contrast effect.

Fixed effects	Estimate	SE	*Z*	*p*
(Intercept)	3.26522	0.21357	15.288	<0.001***
Vowel_contrast/ɐ/−/ε/	−0.54817	0.26749	−2.049	0.040431*
Vowel_contrast/ɐ/−/-ɨ/	0.62047	0.32728	1.896	0.057981
Vowel_contrast/e/−/-ɨ/	0.16798	0.2485	0.676	0.499065
Vowel_contrast/ε/−/e/	−2.77152	0.20055	−13.819	<0.001***
Vowel_contrast/ε/−/-ɨ/	0.66981	0.25551	2.622	0.008754**
Testpost-test	1.1541	0.28617	4.033	<0.001***
Vowel_contrast/ɐ/−/ε/:testpost-test	−0.07276	0.36074	−0.202	0.840148
Vowel_contrast/ɐ/−/-ɨ/:testpost-test	−0.41614	0.39291	−1.059	0.289542
Vowel_contrast/e/−/-ɨ/:testpost-test	−0.60966	0.36252	−1.682	0.092616
Vowel_contrast/ε/−/e/:testpost-test	−1.10914	0.30465	−3.641	<0.001***
Vowel_contrast/ε/−/-ɨ/:testpost-test	0.7562	0.45515	1.661	0.096626

Number of observations: 14208, Participants: 37. AIC = 6988.6, BIC = 7669.2, log-likelihood = −3404.3. R syntax: glmer(accuracy ∼ vowel_contrast × test + (1 + test + vowel_contrast + test × vowel_contrast | participant_ID), family = binomial, data = AXB, glmerControl(optCtrl = list(maxfun = 2e5), optimizer = “nloptwrap”, calc.derivs = FALSE)). Reference level: /ɐ/−/e/, pre-test.

*p ≥ 0.05;

**p ≥ 0.01;

***p ≥ 0.001.

The pairwise comparisons also revealed that participants struggled more with the /ε/−/e/ contrast, compared to the other contrasts. This difficulty is also evident in Table 5 and when we look at mean accuracy for each contrast (in Supplementary material): we observe that it was above 90% in pre-test and post-test, for all contrasts except for /ε/−/e/, which was around 60%. Recall that in the lexical identification post-test, at group level we observed more difficulties with the pseudowords containing the vowels /ε/ and /e/ than for other pseudowords (Figure 9, on the right).

**FIGURE 11:**
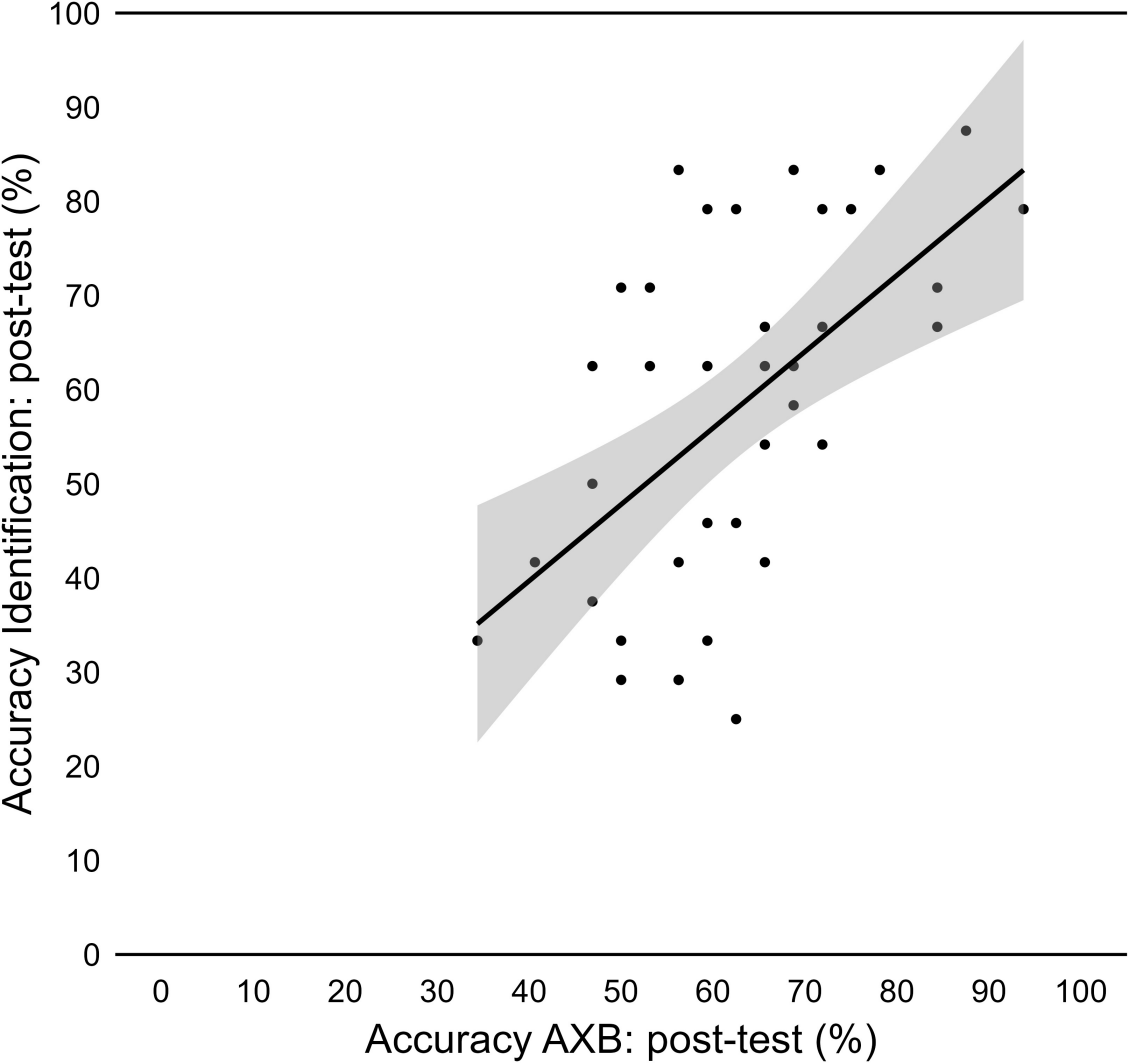
Relationship between performance in the AXB discrimination post-test, for /ε/- /e/, and in the lexical identification post-test, for /gε/, /zε/, /ge/, and/ze/.

To investigate if this link between discrimination and word learning was also present at individual level, we conducted a Pearson’s correlation test with results for the AXB post-test, for the /ε/−/e/ contrast, and results for the identification task, for pseudowords with the vowels /ε/−/e/. The test revealed a positive correlation (*r* = 0.56, *p* < 0.001; Figure 11).

## Discussion

This study aimed to investigate whether learning non-native words contributes to improving the discrimination of non-native vowels. This inquiry is particularly relevant given that identification training tasks are difficult to conduct in naïve participants due to limited or no vocabulary knowledge, and existing research indicates limited success with phonetic training through discrimination tasks at the initial stages of language learning (Correia et al., 2025; Ge et al., 2025a; Tavares, 2024). To this end, we developed a novel CSWL protocol that enhances previous CSWL tasks by incorporating new features, thereby transforming it into an effective training tool.

We trained Hungarian native speakers with eight Portuguese pseudowords, each mapped to a nonsense object, in a CSWL session, consisting of alternate passive and active blocks. In the first, participants observed word-object pairings from sequences of trials with two pseudowords and two objects; in the later, they heard a single word and chose the corresponding object from two options, receiving feedback. Learners completed an eight- alternative forced-choice task before and after training to evaluate word learning. An AXB task was also administered in pre- and post-test to assess changes in discrimination.

The results from the CSWL training indicate that its design was effective: at the end of the first block (i.e., after approximately 10 min), participants achieved an overall accuracy of 87.2% in identifying the eight Portuguese pseudowords, with the lowest individual mean accuracy at 67%. At the end of the training session, those values were 95.0% and 83%, respectively. Although data analysis revealed differences in the vowels’ learning trajectories, all pseudowords were identified significantly above chance during the CSWL and in the identification post-test. Notably, since the lexical identification task was administered the day after training, these results provide strong evidence for retention of learning, further underscoring the robustness of the observed effects.

These results align with findings from Tuninetti et al. (2020) and Escudero et al. (2022) but partially differ from those of Ge et al. (2025a), since in the latter no learning was observed for vowel or consonant minimal pairs. Recall that in the learning phases of the first two studies, as well as in the passive CSWL trials in our study, participants were exposed to contrasts in each trial. However, in Ge et al. (2025a) participants were presented with a single word per trial. Accordingly, our study supports the benefit of training listeners with contrasts presented within the same trial.

We should also highlight three innovations in our CSWL design that may have contributed to its effectiveness. First, the task featured alternating passive and active trials, mirroring real-world language learning environments where learners progress from listening to active practice, through trial and error (Monaghan et al., 2019). Second, providing feedback to participants may have further contributed to the robustness of learning. This interpretation is supported not only by the well-established positive effects of feedback in L2 learning contexts (Li, 2010; Monaghan et al., 2021), but also by direct comparisons with previous CSWL experiments. For instance, Escudero et al. (2022) and Tuninetti et al. (2020) did not provide feedback to participants, and their reported mean accuracy scores were lower than those observed in our study. Conversely, Monaghan et al. (2019) found that the group receiving feedback during training achieved the highest accuracy, further underscoring the beneficial impact of feedback on L2 learning outcomes. Finally, unlike previous CSWL tasks focused on L2 phonology, in our task, tokens were produced by different native speakers (two female Portuguese speakers). This exposure to the same word produced by different speakers may have enhanced robust learning, in line with previous research in vocabulary learning (Barcroft and Sommers, 2005) and phonetic training (Thomson, 2018). Moreover, recent studies suggest that the presence of speaker variability itself—rather than the number of speakers—is beneficial for perceptual learning (Nagle et al., 2025).

### RQ1: Can CSWL improve the discrimination of non-native vowel contrasts?

We predicted that learning novel words through CSWL would lead to measurable improvement in the discrimination of non-native vowel contrasts, especially for contrasts absent from the learners’ L1. This prediction was motivated by research suggesting that lexical learning supports phonological development by helping learners establish new phonological categories through meaningful input (Cutler et al., 2006; Escudero and Williams, 2014). Moreover, exposure to minimal pairs or contrastive contexts has been shown to facilitate perceptual learning (Escudero et al., 2022; Tuninetti et al., 2020).

The results for the AXB tests revealed a significant improvement in three of the six contrasts: /ɐ/−/ε/, /ɐ/−/e/, and /ε/−/-ɨ/. The positive result for the first contrasts is especially important considering that Hungarian speakers struggle to perceive the two Portuguese vowels as separate categories (Tavares et al., 2024). However, the results for the AXB tests should be interpreted with caution, since in the case of five contrasts accuracy was at ceiling (91.5% was the lowest mean accuracy), suggesting that not all contrasts need training. Furthermore, we observe lack of improvement in the discrimination /ε/−/e/, a contrast in which Hungarian speakers performed significantly worse than in the remaining contrasts. In other words, our learners did not benefit from the CSWL training in the contrast where they had the greatest need for improvement.

Difficulties with mid-vowel contrasts are often observed in speakers of languages that lack such distinctions, and even bilinguals who have these distinctions in one of their L1s struggle with these contrasts (Amengual, 2014; Kogan and Tavares, 2024; Mora et al., 2011). Such difficulties were also reported in the perception of Portuguese mid-vowels. For instance, Macedo (2015) found that Canadian English speakers, who were beginner learners of L2 Portuguese, systematically categorized both Portuguese /ε/ and /e/ into the English /ε/. Similarly, Correia et al. (2025) documented challenges in perceptual discrimination: British English participants with no prior knowledge of Portuguese exhibited low accuracy for the /ε/–/e/ contrast, significantly lower than for the /ɔ/–/o/ mid-vowel contrast. However, the participants in the present study partially differ from those in the aforementioned studies. While Hungarian includes both vocalic qualities in its inventory, /ε/ exists as a short vowel and /eː/ as a long vowel (Markó, 2017). In Hungarian, vowel length is a contrastive feature (Markó, 2017; e.g., /’øryl/ ‘rejoiced’ vs. /’ø:ryl/ ‘getting crazy’), making Hungarian listeners particularly sensitive to vowel duration. However, in the auditory stimuli we used in our experiment—retrieved from Tavares (2024)—vowel duration was normalized across tokens. Our participants may have been confused by the similar vowel durations in our tokens. In other words, vowel quality does not serve as the sole acoustic cue for the /ε/–/eː/contrast in Hungarian. By removing the durational contrast, we likely increased the difficulty of discriminating between the two Portuguese vowels. This issue points to the importance of stimuli-specific features, as pointed in Escudero et al. (2022).

### RQ2: Do perceptual difficulties affect word learning?

We predicted that pseudowords containing harder-to-discriminate vowels, such as /ε/ and /e/, would be more difficult to learn. This prediction is supported by research showing that perceptual confusability constrains lexical encoding (Darcy et al., 2024; Best et al., 2001; Trofimovich and John, 2011). When phonetic contrasts are not perceived accurately, learners are more likely to develop overlapping or unstable lexical representations (Cutler et al., 2006; Escudero et al., 2008).

This was confirmed: both in the CSWL training and in the lexical identification test, participants displayed greater difficulties with pseudowords containing these vowels, suggesting a link between difficulties in discrimination and the ability to learn novel words. Furthermore, we found a correlation at the individual level between discrimination performance and word learning success, indicating that participants who struggled more with discrimination of these vowels also had more difficulty learning the corresponding novel words.

Importantly, the results of our AXB tests diverged from those of Tavares et al.’s (2024) oddity discrimination tasks: while participants achieved ceiling accuracy in all but the /ε/−/e/ contrast in our study, the oddity discrimination task revealed Hungarian participants’ difficulties across multiple contrasts. This discrepancy likely stems from AXB’s lower cognitive demands compared to oddity tasks. In an oddity trial, participants must engage in a two-step decision process: first, determining if a deviant token exists (with uncertainty heightened by catch trials), and second, locating it if present. This contrasts with the AXB task, which involves a single decision based on category matching. Additionally, the oddity task’s requirement to identify the deviant token in any of the three positions further complicates the decision-making process, contributing to its increased difficulty. These differences underscore how task selection shapes the detection of perceptual challenges: incorporating an oddity task in the present study could have revealed additional discrimination patterns, thereby clarifying relationships between word learning and perceptual discrimination.

### RQ3: Is CSWL effective for *ab initio* learners?

We predicted that even *ab initio* learners with no prior exposure to the target language would be able to acquire novel pseudowords through a single CSWL session. This prediction builds on findings from statistical and cross-situational learning studies, which demonstrate that learners can rapidly acquire word–referent mappings even with non-native words (Escudero et al., 2022; Ge et al., 2025b,2025c; Tuninetti et al., 2020). The addition of feedback and speaker variability aimed to enhance attention to phonetic detail and support more robust category formation (Barcroft and Sommers, 2005; Thomson, 2018).

This prediction was strongly supported. Participants performed significantly above chance both during training and in the post-training identification task, demonstrating that CSWL is a feasible and effective method for supporting early L2 lexical and perceptual learning. Importantly, mean accuracy in our experiment was higher than in previous studies that used a similar procedure but did not include feedback (Escudero et al., 2022; Tuninetti et al., 2020). This finding further highlights the positive effect of providing feedback to participants.

### RQ4: Do vowel contrasts influence learning outcomes?

We anticipated that pseudowords involving perceptually easier vowel contrasts, such as /ε/−/-ɨ/, would be learned and discriminated more successfully than those containing more challenging contrasts, particularly /ε/–/e/. This prediction reflects research showing that perceptual salience influences phonological and lexical learning outcomes (Escudero and Williams, 2014; Mora et al., 2011). This was confirmed in our findings. Importantly, the correlation between word learning success and discrimination (Figure 11) underscores the interconnected nature of these skills.

Our findings reinforce the theoretical strengths of CSWL highlighted in the literature, demonstrating that this paradigm is not only a valuable tool for observing L2 word learning, but can also serve as an effective training instrument. The flexibility inherent to CSWL allowed us to adapt its conventional application into a robust training protocol by incorporating features such as explicit feedback, speaker variability, and the alternation of observational and active tasks. Notably, our results suggest that CSWL primarily engages implicit learning mechanisms, supporting learners in forming phono-lexical representations without the need for explicit instruction and mirroring naturalistic language learning. Moreover, a significant advantage of CSWL is its suitability for use with *ab initio* learners, as it does not require prior lexical or semantic knowledge. However, the persistent difficulty observed with the most challenging contrast suggests that explicit guidance or targeted instruction may further enhance learning outcomes in particular situations.

### Limitations and further directions

While the results of this study are promising, several limitations must be acknowledged. First, the use of an AXB task may have introduced ceiling effects for most contrasts, limiting our ability to detect more nuanced learning effects. Incorporating more demanding perceptual tasks, such as oddity discrimination with catch trials, may provide greater sensitivity to change in future research.

Second, our vowel stimuli were normalized for duration, which, although methodologically controlled, may have inadvertently hindered performance in contrasts where duration is a relevant cue in the learners’ L1. Future studies should explore the effects of using more naturalistic speech stimuli that preserve variation in duration and other acoustic cues.

Third, this study focused on short-term learning; future research should explore retention over time and the potential for generalization to novel word forms or contexts. Longitudinal studies would provide valuable insights into the durability and scope of CSWL-based training effects.

Fourth, the limited improvement observed for the /ε/–/e/ vowel contrast suggests that certain distinctions may require more targeted or extended training. Future studies should investigate whether increased exposure or focused training on particularly difficult contrasts can facilitate greater perceptual gains in these areas.

Fifth, including a control group of participants exposed to the same auditory stimuli but without referent mapping would strengthen our conclusions, particularly by clarifying whether improvements in discrimination are attributable to the training itself rather than to repeated exposure. To address this, we are currently designing a follow-up experiment that will directly examine this issue.

Finally, future work may also benefit from examining individual differences in learning outcomes, including cognitive, perceptual, and motivational factors, which could influence how learners respond to CSWL tasks. A more nuanced understanding of learner variability will be essential for tailoring perceptual training methods to diverse learner profiles.

## Conclusion

This study investigated whether CSWL can serve as an effective perceptual training tool for adult *ab initio* learners of an L2. Specifically, we examined whether learning novel words that contain non-native vowels supports improved discrimination of those same vowels. Hungarian participants learned eight Portuguese pseudowords mapped to novel objects in a CSWL session combining passive and active learning, speaker variability, and trial-by-trial feedback. Post-training results from a forced-choice lexical identification task showed that participants learned all pseudowords significantly above chance. However, accuracy varied by vowel: pseudowords with /ε/ or /e/ were identified less accurately than those with /-ɨ/ or /ɐ/, indicating that perceptual difficulty constrained word learning outcomes.

Discrimination results further supported this pattern. While participants improved in three of six vowel contrasts, notably including vowels absent from their L1, no improvement was observed for /ε/–/e/, the contrast perceived as most difficult. Importantly, individual performance in discriminating this contrast was positively correlated with word learning success, underscoring the close relationship between perceptual acuity and lexical development. Furthermore, the AXB task used to assess discrimination yielded near-ceiling performance for most contrasts, limiting its sensitivity to training effects. To address this limitation, a second experiment is underway incorporating both AXB and oddity tasks with catch trials, which may better reveal perceptual learning trajectories.

Overall, our findings highlight CSWL’s promise as a meaning-oriented method for supporting early phonological learning. The results suggest that embedding difficult non-native contrasts in lexically meaningful contexts can promote learning, though some contrasts may require more targeted support. This has practical implications for L2 instruction and theoretical implications for research on the cognitive mechanisms underlying language learning and speech perception. Future studies should explore the role of task design and individual differences in optimizing CSWL for perceptual training across learner profiles and linguistic contexts.

## Data Availability

The datasets presented in this study can be found in online repositories. The names of the repository/repositories and accession number(s) can be found below: https://osf.io/ey8tf/?view_only=412353eda8f24bc6ba0be3bdad58b60f.
