# Supplementary material for: From meaning to sound: how word learning shapes non-native speech perception
Source: Front Psychol. 2025 Sep 5;16:1620837. doi: 10.3389/fpsyg.2025.1620837 (PMC12447442; doi:10.3389/fpsyg.2025.1620837)
Supplement: Supplementary file 1 [file Table_1.docx]

Supplementary Material

**Supplementary Table 1**. List of the four word-object mappings

|  |  | Nonsense objects | | | |
| --- | --- | --- | --- | --- | --- |
|  |  | Mapping 1 | Mapping 2 | Mapping 3 | Mapping 4 |
| Pseudowords | /ɡɐ/ | A | H | G | F |
|  | /gɛ/ | B | A | H | G |
|  | /ɡe/ | C | B | A | H |
|  | /gɨ/ | D | C | B | A |
|  | /zɐ/ | E | D | C | B |
|  | /zɛ/ | F | E | D | C |
|  | /ze/ | G | F | E | D |
|  | /zɨ/ | H | G | F | E |

**Supplementary Table 2**. List of the contrasts by type

| Vowel minimal pairs  (n = 12) | /ɡɐ/−/ɡɛ/, /ɡɐ/−/ɡe/, /ɡɐ/−/ɡɨ/, /ɡɛ/−/ɡe/, /ɡɛ/−/ɡɨ/, /ɡe/−/ɡɨ/ |
| --- | --- |
|  | /zɐ/−/zɛ/, /zɐ/−/ze/, /zɐ/−/zɨ/, /zɛ/−/ze/, /zɛ/−/zɨ/, /ze/−/zɨ/ |
| Consonant minimal pairs  (n = 4) | /ɡɐ/−/zɐ/, /ɡɛ/−/zɛ/, /ɡe/−/ze/, /ɡɨ/−/zɨ/ |
| Non-minimal pairs  (n =12) | /ɡɐ/−/zɛ/, /ɡɐ/−/ze/, /ɡɐ/−/zɨ/, /ɡɛ/−/ze/, /ɡɛ/−/zɨ/, /ɡe/−/zɨ/ |
|  | /zɐ/−/ɡɛ/, /zɐ/−/ɡe/, /zɐ/−/ɡɨ/, /zɛ/−/ɡe/, /zɛ/−/ɡɨ/, /ze/−/ɡɨ/ |

**Supplementary Table 3.** Balance between blocks, by condition, for the CSWL *passive* task

|  | Total of trials | Number of trials by block |
| --- | --- | --- |
| Number of trials | 224 | 56 |
| Occurrences of each pseudoword first | 28 | 7 |
| Occurrences of each pseudoword secondly | 28 | 7 |
| Occurrences of each object on the left | 28 | 7 |
| Occurrences of each object on the right | 28 | 7 |
| Vowel contrast doublets | 96 | 24 |
| Consonant contrast doublets | 32 | 8 |
| Non-minimal pair doublets | 96 | 24 |
| Occurrences of speaker 1 | 112 | 28 |
| Occurrences of speaker 2 | 112 | 28 |
| Occurrences of congruent order | 112 | 28 |
| Occurrences of incongruent order | 112 | 28 |

**Supplementary Table 4.** Balance between blocks, by condition, for the CSWL *active* task

|  | Total of trials | Number of trials by block |
| --- | --- | --- |
| Number of trials | 224 | 56 |
| Occurrences of each pseudoword | 28 | 7 |
| Occurrences of each object on the left | 28 | 7 |
| Occurrences of each object on the left | 28 | 7 |
| Vowel contrast doublets | 96 | 24 |
| Consonant contrast doublets | 32 | 8 |
| Non-minimal pair doublets | 96 | 24 |
| Occurrences of speaker 1 | 112 | 28 |
| Occurrences of speaker 2 | 112 | 28 |
| Answers corresponding to object on the right | 112 | 28 |
| Answers corresponding to object on the left | 112 | 28 |

**Supplementary Table 5.** Results for the *active* CSWL training trials, by block

| block | N | accuracy | SD | SE | CI |
| --- | --- | --- | --- | --- | --- |
| Block 1 | 2016 | 87.70 | 37.94 | 0.84 | 1.66 |
| Block 2 | 2072 | 92.57 | 30.29 | 0.67 | 1.31 |
| Block 3 | 2072 | 93.34 | 28.80 | 0.63 | 1.24 |
| Block 4 | 2072 | 94.98 | 25.22 | 0.55 | 1.09 |

**Supplementary Table 6.** Results for the *active* CSWL training trials as a function of vowel, by block

| vowel | block | N | accuracy | SD | SE | CI |
| --- | --- | --- | --- | --- | --- | --- |
| /ɐ/ | Block 1 | 504 | 91.27 | 29.18 | 1.30 | 2.55 |
|  | Block 2 | 518 | 94.79 | 22.98 | 1.01 | 1.98 |
|  | Block 3 | 518 | 95.17 | 22.16 | 0.97 | 1.91 |
|  | Block 4 | 518 | 95.75 | 20.85 | 0.92 | 1.80 |
| /e/ | Block 1 | 518 | 85.52 | 36.38 | 1.60 | 3.14 |
|  | Block 2 | 518 | 92.66 | 26.95 | 1.18 | 2.33 |
|  | Block 3 | 518 | 87.64 | 34.02 | 1.49 | 2.94 |
|  | Block 4 | 518 | 92.86 | 26.62 | 1.17 | 2.30 |
| /ɛ/ | Block 1 | 518 | 83.20 | 38.65 | 1.70 | 3.34 |
|  | Block 2 | 518 | 87.64 | 34.02 | 1.49 | 2.94 |
|  | Block 3 | 518 | 92.66 | 26.95 | 1.18 | 2.33 |
|  | Block 4 | 518 | 93.24 | 25.95 | 1.14 | 2.24 |
| /ɨ/ | Block 1 | 518 | 89.38 | 31.85 | 1.40 | 2.75 |
|  | Block 2 | 518 | 95.17 | 22.16 | 0.97 | 1.91 |
|  | Block 3 | 518 | 97.88 | 14.90 | 0.65 | 1.29 |
|  | Block 4 | 518 | 98.07 | 14.22 | 0.62 | 1.23 |

**Supplementary Table 7.** Results for the lexical identification trials, by test

| test | N | accuracy | SD | SE | CI |
| --- | --- | --- | --- | --- | --- |
| pretest | 1776 | 13.57 | 48.45 | 1.15 | 2.25 |
| post-test | 1776 | 71.17 | 64.08 | 1.52 | 2.98 |

**Supplementary Table 8.** Results for the lexical identification trials as a function of vowel, by test

| vowel | test | N | accuracy | SD | SE | CI |
| --- | --- | --- | --- | --- | --- | --- |
| /ɐ/ | pretest | 444 | 14.41 | 37.59 | 1.78 | 3.51 |
|  | post-test | 444 | 80.41 | 42.48 | 2.02 | 3.96 |
| /e/ | pretest | 444 | 14.86 | 38.07 | 1.81 | 3.55 |
|  | post-test | 444 | 54.73 | 53.27 | 2.53 | 4.97 |
| /ɛ/ | pretest | 444 | 13.96 | 37.10 | 1.76 | 3.46 |
|  | post-test | 444 | 61.04 | 52.19 | 2.48 | 4.87 |
| /ɨ/ | pretest | 444 | 11.04 | 33.54 | 1.59 | 3.13 |
|  | post-test | 444 | 88.51 | 34.13 | 1.62 | 3.18 |

**Supplementary Table 9.** Results for the AXB trials, by test

| test | N | accuracy | SD | SE | CI |
| --- | --- | --- | --- | --- | --- |
| pretest | 7104 | 88.92 | 44.39 | 0.53 | 1.03 |
| post-test | 7104 | 90.82 | 40.83 | 0.48 | 0.95 |

**Supplementary Table 10.** Results for the AXB trials, as a function of vowel contrast, by test

| vowel contrast | test | N | accuracy | SD | SE | CI |
| --- | --- | --- | --- | --- | --- | --- |
| /ɐ/−/e/ | pretest | 1184 | 94.51 | 23.80 | 0.69 | 1.36 |
|  | post-test | 1184 | 96.37 | 19.55 | 0.57 | 1.11 |
| /ɐ/−/ɛ/ | pretest | 1184 | 91.47 | 29.19 | 0.85 | 1.66 |
|  | post-test | 1184 | 94.93 | 22.92 | 0.67 | 1.31 |
| /ɐ/−/ɨ/ | pretest | 1184 | 95.27 | 22.18 | 0.64 | 1.26 |
|  | post-test | 1184 | 97.80 | 15.31 | 0.45 | 0.87 |
| /e/−/ɨ/ | pretest | 1184 | 94.09 | 24.64 | 0.72 | 1.41 |
|  | post-test | 1184 | 96.03 | 20.40 | 0.59 | 1.16 |
| /ɛ/−/e/ | pretest | 1184 | 61.74 | 50.78 | 1.48 | 2.90 |
|  | post-test | 1184 | 62.42 | 50.61 | 1.47 | 2.89 |
| /ɛ/−/ɨ/ | pretest | 1184 | 96.45 | 19.33 | 0.56 | 1.10 |
|  | post-test | 1184 | 97.38 | 16.68 | 0.48 | 0.95 |
